# How many people have had a myocardial infarction? Prevalence estimated using historical hospital data

**DOI:** 10.1186/1471-2458-7-174

**Published:** 2007-07-24

**Authors:** Douglas G Manuel, Jenny JY Lim, Peter Tanuseputro, Therésè A Stukel

**Affiliations:** 1 Institute for Clinical Evaluative Sciences, G106-2075 Bayview Avenue, Toronto, Ontario, M4N 3M5, Canada; 2 Department of Public Health Sciences, University of Toronto, Toronto, Canada; 3 Department of Health Policy Management and Evaluation, University of Toronto, Toronto, Canada

## Abstract

**Background:**

Health administrative data are increasingly used to examine disease occurrence. However, health administrative data are typically available for a limited number of years – posing challenges for estimating disease prevalence and incidence. The objective of this study is to estimate the prevalence of people previously hospitalized with an acute myocardial infarction (AMI) using 17 years of hospital data and to create a registry of people with myocardial infarction.

**Methods:**

Myocardial infarction prevalence in Ontario 2004 was estimated using four methods: 1) observed hospital admissions from 1988 to 2004; 2) observed (1988 to 2004) and extrapolated unobserved events (prior to 1988) using a "back tracing" method using Poisson models; 3) DisMod incidence-prevalence-mortality model; 4) self-reported heart disease from the population-based Canadian Community Health Survey (CCHS) in 2000/2001. Individual respondents of the CCHS were individually linked to hospital discharge records to examine the agreement between self-report and hospital AMI admission.

**Results:**

170,061 Ontario residents who were alive on March 31, 2004, and over age 20 years survived an AMI hospital admission between 1988 to 2004 (cumulative incidence 1.8%). This estimate increased to 2.03% (95% CI 2.01 to 2.05) after adding extrapolated cases that likely occurred before 1988. The estimated prevalence appeared stable with 5 to 10 years of historic hospital data. All 17 years of data were needed to create a reasonably complete registry (90% of estimated prevalent cases). The estimated prevalence using both DisMod and self-reported "heart attack" was higher (2.5% and 2.7% respectively). There was poor agreement between self-reported "heart attack" and the likelihood of having an observed AMI admission (sensitivity = 63.5%, positive predictive value = 54.3%).

**Conclusion:**

Estimating myocardial infarction prevalence using a limited number of years of hospital data is feasible, and validity increases when unobserved events are added to observed events. The "back tracing" method is simple, reliable, and produces a myocardial infarction registry with high estimated "completeness" for jurisdictions with linked hospital data.

## Background

A cornerstone of population health is the estimation of disease occurrence – both in terms of incidence (people who newly develop a disease) and prevalence (the total number of people living with a disease). Disease occurrence is routinely estimated using three methods. The two most common are disease registries using active case identification (e.g. cancer registries) and health surveys that ask people whether they have a disease. Health surveys are either designed for specific diseases/conditions, or they simultaneously gather information about several diseases/conditions, along with other health status measures and behaviours (e.g. smoking).

A third method, the focus of this study, uses routinely collected health administrative data such as hospital discharge data. Wider availability of health administrative data has, in part, contributed to more widespread use of these data for measuring disease occurrence. However, health administrative data are often not available over long time periods, which has made it difficult to distinguish people who newly develop a chronic disease from those who have had the disease for a long time. Furthermore, health administrative data that are individually linked across time and to different data sources have increasingly been used to create registries of people with chronic diseases. However, the registries do not include people whose most recent event happened before the availability of data.

This study seeks to demonstrate that hospital data can be reliably used to estimate the number of people in Ontario, Canada, who have ever been admitted to a hospital with an acute myocardial infarction (AMI). We also demonstrate that hospital data can be used to create a registry of people with a myocardial infraction (MI). By examining MI, we hope to better understand when health administrative data can be used to estimate disease prevalence and to develop disease registries for other conditions.

Health administrative data are commonly used to measure disease occurrence. For the past five years in Canada, the primary source of information on diabetes occurrence has been the National Diabetes Surveillance System (NDSS), using physician outpatient service and hospital discharge data[1,2] Survival, migration and other demographic information are obtained by linking people with diabetes to health care eligibility registries – which include virtually all Canadians. Similar examples include the Canadian Congenital Abnormality Surveillance System[3], the Canadian Stroke Network[4], and, within specific provinces, diseases/conditions such as injury[5], congestive heart failure[6], and asthma[7]. Internationally, there are more examples.

While commonly used to identify disease events, health administrative data are rarely used to estimate disease prevalence or to create registries, with the exception of surveillance for two types of chronic diseases. The first type is conditions such as diabetes that require frequent and continuous medical care, so that prevalent cases can be estimated with just a few years of data. The second type is conditions that are identified by acute manifestation of a chronic disease and whose survival is low, such as congestive heart failure,[8] where only a few years of observed hospital events (acute exacerbation) and mortality data are needed to reliably estimate the number of people currently living with the condition (prevalent cases).

AMI is unlike either of these two types of diseases. AMI is an acute manifestation of coronary heart disease with events usually separated by several years[9] Compared to congestive heart failure, survival is more favorable (78% one-year AMI survival in Ontario)[10].

The successful use of health administrative data to estimate prevalence and create disease registries for diabetes and congestive heart failure has encouraged us to examine whether health administrative data can be used in more challenging settings such as MI. Specific objectives of this study are to: 1) estimate MI prevalence in 2004 using 17 years of hospital data (1988 to 2004); 2) create a registry of people with an MI using the same data; 3) examine how different periods of hospital discharge data (5, 10, 15 years) affect the reliability of prevalence estimates and the registry's "completeness" (defined as the number people in the registry compared to the estimated total number of people previously hospitalized for AMI)[11]; and 4) compare these prevalence estimates with other commonly used methods, such as DisMod software and a population health survey.

## Methods

### MI registry and prevalence populations

People were included into the MI registry if they had an AMI hospitalization between 1988 and 2004 and, at the time of the AMI event, were Ontario residents, age 20 to 105, and eligible for health care coverage (almost all residents).

To estimate MI prevalence in 2004 for the health administration method, we identified people in the MI registry who were: alive for any time during 2004; eligible for health care for any time during 2004; and used health care at least once during 2002 or 2003 (to further exclude people who were likely no longer Ontario residents). To compute MI prevalence rates for 2004, adjusted population estimates by Statistics Canada were used to calculate the age-specific Ontario resident population. DisMod used the identical MI registry and population estimates. Self-reported "heart attack" prevalence was estimated from the Canadian Community Health Survey (CCHS), which reflects community-dwelling Ontario residents in 2000/01 (see below for details).

### Data sources and AMI identification

#### Determining hospitalized AMI events using health administrative data

People with a hospital admission for AMI were identified using the Discharge Abstract Database (DAD) of the Canadian Institute for Health Information (CIHI). The CIHI DAD contains demographic, administrative and clinical information for all Ontario hospital admissions since April 1, 1988.

AMI hospitalization was defined as any admission between fiscal year 1988 to 2004 using International Classification of Diseases (ICD) codes 410 (9^th ^revision) or I21, I22 and I51.3 (10^th ^revision). Coding accuracy of AMI diagnosis using hospital discharge data has been validated through multicentre chart audits (sensitivity 88.8%, specificity 92.8% for patients admitted to coronary care units)[12-14] Admissions were excluded if the hospital stay was less than four days for those discharged alive or if the patient was transferred from another acute care facility. Complete details and rationale for the inclusion and exclusion criteria have been reported previously[7].

People with an AMI admission were deterministically linked using unique encrypted health card numbers to the Ontario Registered Persons Database, which provided data on mortality over time, regardless of where death occurred, as well as periods of health care eligibility and health care use (physician visit, drug prescription, hospital or emergency room visit).

#### Estimating self-reported "heart attack" from a population health survey

People with a self-reported "heart attack" were determined using the Canadian Community Health Survey 1.1 (CCHS 1.1), a national population-based health survey, conducted by Statistics Canada between September 2000 and November 2001[15] Residents of Indian reserves, long-term care institutions, prisons, and remote areas, as well as Foreign Service personnel, were excluded. The 34,189 Ontario respondents over the age of 20 years were asked for permission to link their responses to their health administrative data (success rate 83.2%)[16] All respondents were asked if they had heart disease diagnosed by a physician and, if so, if they ever had a heart attack. The respondents whose survey data were individually linked to their CIHI DAD data were used to assess inter-database reliability.

#### Data for DisMod

The DisMod software, offered by World Health Organization, uses life-table or stationary population technique to indirectly estimate the prevalence of a disease from the most recent years of observed incidence and mortality data[17] DisMod requires data on age-specific AMI incidence rates, cause-specific mortality rates, and population counts. CIHI DAD discharge records, as previous defined, were used to estimate AMI incidence rates. All-cause and MI specific mortality were from the Canadian Mortality Database, Statistics Canada, for years 2001 to 2003.

### Estimating prevalence

#### Prevalence using hospital discharge data

For Ontarians who were alive at the end of 2004 and had a valid health card, we first counted the number of people who had at least one hospital admission for MI from 1988 to 2004 ("observed events"). Then we added the estimated number of people with an admission between 1920 and 1987 ("unobserved events" for the time period with no available hospital data) in three steps. First, we identified the most recent year of AMI hospitalization from 2004 back to 1988. This is referred to as "back tracing" events, which is the same as a time-to-event or survival analysis – except that the events are backward in time, instead of the more typical approach of estimating events looking forward in time[18]. These observed events were grouped and plotted by year and sex for the age groups 20–49, 50–64, 65–79, and 80 + years. The second step assumed that the yearly observed event rates from 2004 to 1988 followed a Poisson distribution and used maximum likelihood methods to predict the mean numbers of annual unobserved events from 1987 to 1920 using the model,

ln *C*(*T *- *t*) = *α *+ *β*_1 _*(2004 - *t*) + *β*_2 _*(2004 - *t*)^2^, t = y ears and T = 2004, where C(T-t) is the count of events.

The third step added the predicted annual unobserved events for the years 1987 to 1920 (C_u_), derived from the model, to the observed events for the period 2004 to 1988, thereby estimating the overall prevalence rate for the Ontario population, 2004.

In sensitivity analyses, we estimated the total prevalence rate with fewer years of observed data and different prediction formulas such as linear and exponential Poisson models.

#### Prevalence using DisMod

Prevalence using DisMod was estimated by inputting into the software by 5-year age groups: AMI incidence rates; all-cause and AMI-specific mortality rates; and population counts. The most current AMI incident rate was estimated by identifying people with a hospital admission in 2004 and no AMI admission from 1988 to 2003.

#### Self-reported "heart attack."

The third method of estimating AMI prevalence used the Ontario sample of the CCHS to obtain people with self-reported "heart attack" by age groups.

Standard errors for unobserved hospital AMI admissions were estimated from the predicted regression model. Bootstrapping methods were used for self-reported "heart attack." [19]

## Results

**Figure 1:**
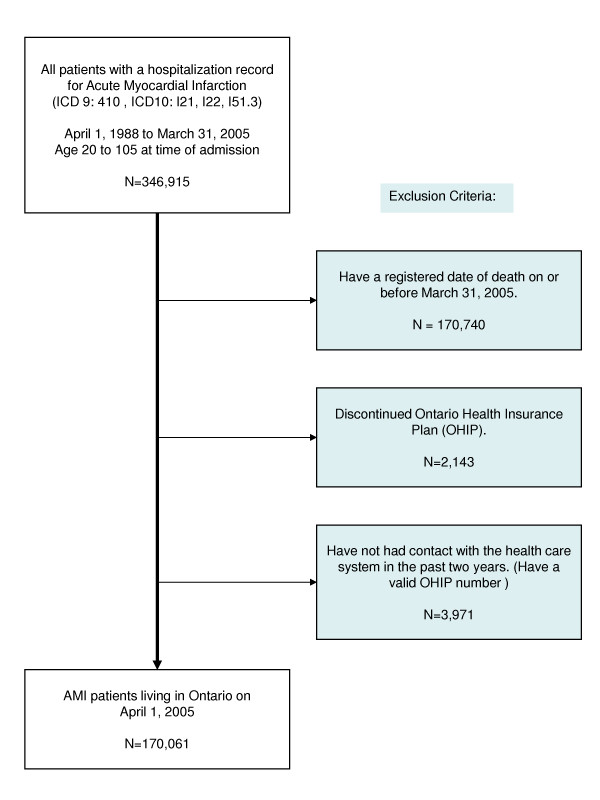
Defining Myocardial Infarction to estimate its prevalence in Ontario, 2004.

The total number of adult Ontarians with at least one AMI hospital admission during 1988–2004 was 346,915. Of these people, 170,061 were alive in 2004 (1.8% of the adult population). The remaining 170,740 people either died (N = 170,740), emigrated (N = 2,143) or had no recent contact with the health care system, likely because they had emigrated or had an invalid health card (N = 3,971) (Figure 1). Table 1 shows the characteristics of those with an AMI hospitalization. Mean period of follow-up is less than 17 years (14.1 years), in part reflecting immigration into Ontario from other countries. The current yearly immigration rate exceeds 1% of the total population and about 30% of residents were born outside Ontario.

**Table 1:** Characteristics of people living in Ontario, 2004 with a hospitalization for acute myocardial infarction, Ontario 1988 to 2004.

		Men	Women
Ontario Population		4,526,367	4,750,578
AMI Prevalence (%)		2.5	1.2

Mean Age		67.4	74.0
Age (%)			
	20–49	8.3	4.1
	50–64	34.6	18.7
	65–79	41.4	41.2
	80+	15.7	36.0
# of years with valid health insurace			
	0–5	0.5	0.4
	6–10	1.7	1.3
	11–15	6.1	4.3
	15+	91.8	93.9
# of years with AMI admissions (%)			
	1	88.4	89.3
	2	10.0	9.1
	3+	1.7	1.7

Data Source: Statistics Canada, Hospital Discharge Abstract Database

**Figure 2:**
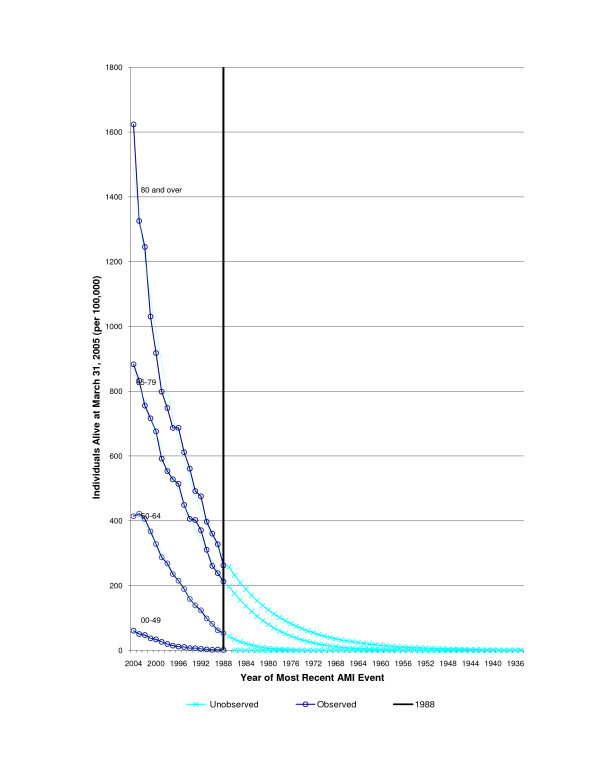
Acute Myocardial Infarction events by most current hospitalization year, in Males alive in Ontario, 2004.

**Figure 3:**
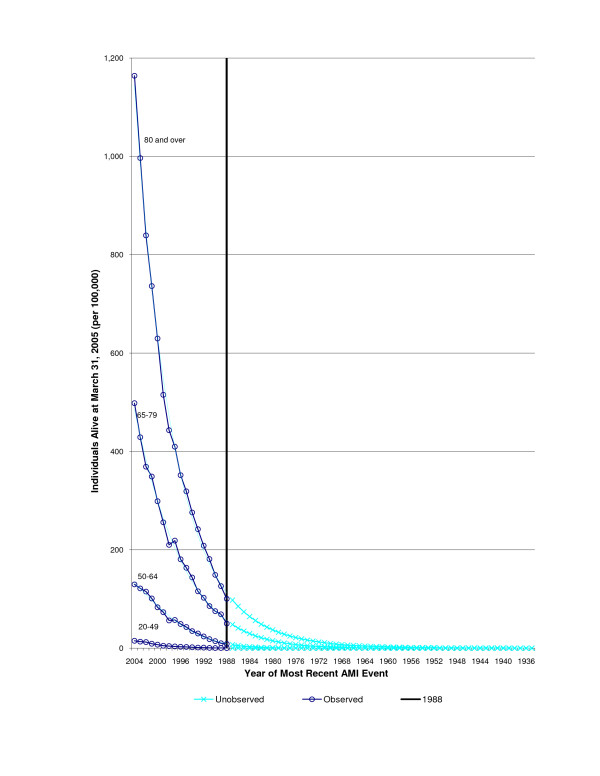
Acute Myocardial Infarction events by most current hospitalization year, in Females alive in Ontario, 2004.

Figures 2 and 3 shows both the number of observed AMI hospitalizations between 1988 and 2004 and the extrapolated (unobserved) AMI admissions from 1920 to 1988 based on Poisson model, by age and sex (Pseudo R^2 ^= 1.00 for all curves). For the observed AMI hospitalizations, there is a rapid decrease in the number of additional people identified with an AMI admission, as one looks further back in time.

The estimated prevalence of MI, combining both observed and unobserved events, was 2.03% (95% confidence interval [95%CI] 2.01 to 2.05%), or 188,000 people. The "completeness" ratio is defined as the number of people with observed AMI admission compared to the total number of observed and unobserved admissions[11] 18,300 (95%CI 16,100 to 20,000) or 10% (95%CI 8.6% to 10.5%) additional people are estimated to have had an AMI prior to 1988, giving a completeness ratio of 0.90. Most of the people with unobserved events were male and older, reflecting higher AMI hospitalization rates for men at younger ages (completeness ratio for men 0.89 versus 0.93 for women).

**Figure 4:**
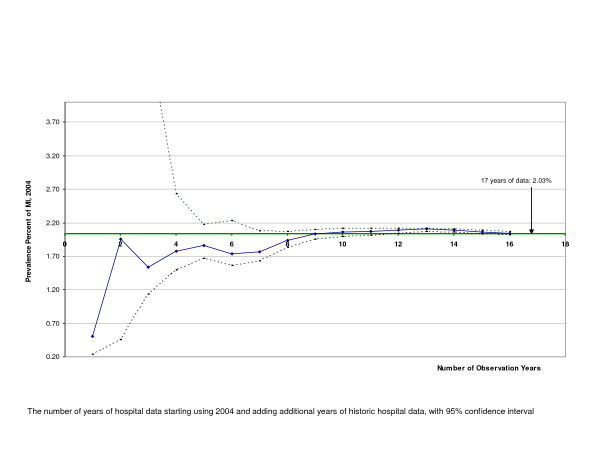
Prevalence of AMI estimated using different number of observational years Caption: The number of years of hospital data starting with 2004 and adding additional years of historic hospital data, with 95% confidence interval.

**Figure 5:**
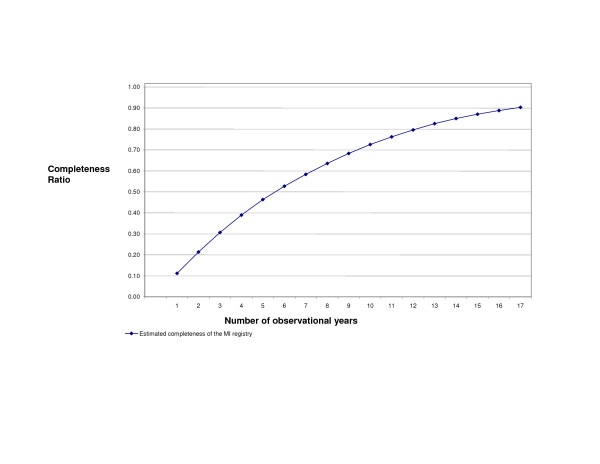
Completeness of the MI Registry, 1 to 17 years of observational years Caption: The completeness of the MI registry.

The estimated prevalence appears reliable and stable after 5 to 10 years of data (Figure 4). The estimated prevalence changed very little if we used different Poisson models for extrapolation (Pseudo R^2 ^greater than 0.98 for all methods, not shown). Figure 5 plots the completeness ratio of the registry with increasing number of years of hospital data. With 5 years of hospital data, the completeness was 47%, slowly leveling off but continually increasing (90% at 17 years of data).

Finally, we estimated the prevalence of MI using the DisMod approach and self-reported "heart attack" from a population-based survey (Table 2). Both methods had modestly higher prevalence estimates (2.5% and 2.7%, respectfully); 1.2-fold higher (or 0.5% difference) for the DisMod approach and 1.3 (or 0.7% difference) for the self-reported estimate. Table 3 shows that there is poor agreement between self-report of "heart attack" and the likelihood of AMI hospitalization (sensitivity = 63.5%).

**Table 2:** Comparing MI prevalence* in the adult population (age 20+), Ontario, Canada

Method	Source of data	Males	Females	Both Sexes
1. Observed Cumulative Prevalence from health services cohort	88-04 Hospital DAD & 88-04 RPDB (Mortality)	2.53	1.17	1.83
2. Prevalence from simple mathematical model from AMI cohort data	88-04 Hospital DAD, 88-04 RPDB (Mortality)	2.84 (2.76, 2.93)	1.26 (1.22, 1.31)	2.03 (2.01, 2.05)
3. Prevalence obtained using a life-table, disease simulation software (DisMod)	2004 Hospital DAD (Incidence), 2000 Vital Stats (Mortality), 2004 Stats Can (Ontario Population Structure)	3.22	1.75	2.47
4. Prevalence estimate of self-reported population health survey (CCHS)†	CCHS 1.1, 2000–01, Public Use Microdata File	3.13 (2.95, 3.72)	2.05 (1.69, 2.34)	2.58 (2.41, 2.90)

*Age-Sex Standardized to the 2004 Ontario Population;

† The 95% percent confidence intervals were calculated using the bootstrap program for the linked CCHS 1.1 file

**Table 3:** Comparing Prevalence of Hospitalized Myocardial Infarction in Ontario, 1988 to 2001, data vs. self-reported 'Heart Attack' in the 2000/01 Canadian Community Health Survey

			**Hospital record for Acute Myocardial Infarction ?**	
			**Yes**	**No**

**Self reported heart attack in CCHS**	**Yes**	N (sample)	116,361 (536)	97,899 (431)
		weighted %	1.35	1.13
	**No**	N (sample)	66,945 (278)	8,355,870 (27,217)
		weighted %	0.78	96.74

Total hospitalized with Acute Myocardial Infarction: 2.12%;

Total self-reported heart attack: 2.49%;

Sensitivity: 63.5%; Specificity: 98.6%; Positive Predictive Value: 54.3%

## Discussion

In this study we used routinely collected hospital data to estimate that 2.0% of people living in Ontario in 2004 have previously had an AMI hospital admission. We also created a registry of 346,915 individual people with known previous MI, of whom 170,061 people were alive and living in Ontario in 2004.

This study demonstrates two strengths for the use of hospital data to identify AMI events. First, the predicted prevalence estimates appear reliable, chiefly reflecting the rapid and consistent decrease in the number of newly identified events as the number of years of hospitalized data increases. We used 17 years of hospital data, but prevalence estimates would be almost as reliable if fewer years of hospital data were used (e.g. 5 to 10 years). Other studies assumed that only 5 years of data were needed to distinguish an AMI incidence case from a prevalent case[20,21]. However, over half of Ontarians who were hospitalized with an AMI with no AMI hospitalization in the previous 5 years – what previous investigators would label an "incident" case – had an earlier AMI hospitalization, which means they are actually a prevalent case.

Second, 17 years of data can be used to create a reasonably complete registry, one that contains 90% of people in Ontario in 2004 who have ever been hospitalized for an AMI. The appropriate level of completeness will probably vary with specific applications and the degree to which people in the registry reflect the total MI population. For the 346,915 people in the MI registry, the registry contains information as to the years in which they had an AMI hospitalization, if and when they died, and if and when they migrated to/from Ontario. People in the MI registry can be individually linked to other disease registries (diabetes, CHF, cancer, etc.), as well as to demographic, health status and health care data (physician services, drug prescriptions, home care, long-term care, other hospitalizations, etc.). Amongst other uses, these linked data can be used to examine health risk factors,[22] outcomes such as life and health expectancy[23] and access, effectiveness, and equity of health care and health policy[9,24-27]

Compared to MI prevalence estimated using observed health administrative data plus unobserved extrapolated cases, both DisMod and self-reported estimates of MI were moderately higher. DisMod's uses the same observed health administrative data, but models prevalence using the assumption of a stationary population. This implies that over time there has been no change in the incidence of MI, life expectancy of the population, or the survival of people with MI compared to those without MI. DisMod's estimates for MI will thus be biased because all of these factors have changed in Ontario. DisMod's method remains helpful in many settings: it is easy to calculate and does not require linked health care data. However, DisMod should be used with caution when there are large changes in disease incidence, survival or life expectancy. Because self-reports often underestimate chronic disease prevalence, we were surprised by the higher self-reported "heart attack" estimates in our study [28-30]. People may have difficulty differentiating a diagnosis of a "heart attack" (AMI) from other types of heart disease.

Differences in the study populations among methods will also contribute to differences in prevalence estimates. For example, the CCHS sampled only community-dwelling people. Self-reported MI prevalence estimates would be higher if the CCHS also included the Ontario institutional population, since this population has more chronic conditions compared to people in the community. Disease prevalence estimates are commonly reported for general populations, when the data actually reflect differences from the (implicit) general population. So, differing study populations is a common problem when comparing different prevalence estimates. The MI registry closely reflects the actual Ontario resident population.

Our method is the simplest available approach to estimate MI prevalence when individually linked events, but limited years of data, are available. Estimates are improved by including extrapolated unobserved events. We used Capocaccia and Angelis' concept of prevalence "completeness," which is also similar to the approach by Brameld et al. of "back tracing" data to estimate disease incidence[11,18]. "Back tracing" is time-to-event or survival analyses, except the events are "backward" or historic in time. The Ontario population is open, meaning that there are births, deaths, immigration and migration. The usual "forward" time-to-event approach, excludes people from prevalence estimates if they die or emigrate. In the "backward" approach, people are excluded at birth (event at age 20 years in this study) or prior to immigration into Ontario. By definition, deaths and emigration are not a concern in the backward time-to-event analyses. AMI events are only ascertained for people who were alive and living in Ontario in 2004.

In the present study, "completeness" was used both to estimate prevalence and to characterize the MI registry. Capocaccia and Angelis sought to estimate prevalence completeness for different time periods of observation when survival and incidence rates are known. Our method has the advantage of not specifically requiring information on survival, disease incidence, or other factors like migration and repeated events; the latter may be important when estimating completeness for high-migration populations like Ontario and conditions like MI. Brameld et al. estimated disease incidence using the proportion of newly identified cases that are truly new (incident) cases. Our measure of completeness can be similarly used to easily and reliably estimate MI incidence. With a completeness of 90% in 2004, a newly observed AMI hospitalization in 2005 has about a 90% likelihood of being a first admission (in other words, an incident case).

Most of the limitations in estimating MI prevalence by means of hospital data are neither unique to this method nor easily addressable. There are three main limitations that reflect: 1) the relatively narrow scope of disease identified using hospitalized AMI; 2) changes to AMI diagnosis and classification; and, 3) reliance on extrapolating unobserved events.

First, not all people who have an AMI are admitted to hospital. Some people with symptoms do not go to hospital, while others with a "silent AMI" may not have symptoms. Of further note, hospitalization data does not identify the 1/3 of people with an incident AMI who die before reaching hospital (of course these people do not become prevalent cases)[31] The DisMod method of prevalence estimation has the same limitations when hospital discharge data are used. Additional AMI events (not hospitalized) can be identified by using diagnostic tests such as electrocardiograms (ECGs). Thus, self-reported "heart attack" may include some people who were not hospitalized, but had these tests. However, our linkage between hospital data and self-reports indicates that 36% (0.78/2.12) of people who had an AMI did not report having a "heart attack," raising concerns about the reliability of self-reporting MI. The use of a population-based survey with an examination that includes an ECG is probably the most robust way of estimating MI prevalence. However, this method is costly and will also miss the rising proportion of MIs that do not have ECG changes[32] For stroke incidence, Feigin has advocated the use of multiple data sources both to ensure the identification of non-hospitalized stroke and to validate case ascertainment from any one source of data[33] A similar approach for MI would be appropriate.

Second, the diagnosis and classification of MI and coronary heart disease has changed, and this affects most prevalence estimation methods. The use of more sensitive diagnostic tests such as blood troponin-I levels has increased the number of "mild" AMI events and events that do not have lasting ECG findings, typically classified by clinicians as "non-ST elevation MI" or "non-Q-wave MI"[32] Furthermore, changes from ICD9 to ICD10 (2002 in Ontario) may further affect MI estimates. Our prevalence estimate reflects AMI diagnosis and classification at the time of a person's most recent AMI event.

The third limitation of our method is the reliance on extrapolating events prior to available hospital data. The clear and consistent pattern of diminishing AMI events over time was reassuring. Additional (or fewer) years of data would have a small effect on our prevalence estimates. However, we did not specifically examine the degree to which changes in demography and disease process influence our prevalence estimates, for both observed and extrapolated cases. More complex population models start with a historic population and simulate how many cases will develop over time, given changing disease and demographic factors[34,35] The extra work required to create such models would likely not improve prevalence estimates compared to our approach, in part because complex disease models also have assumptions due to missing historic disease and demographic information (incidence, population structure, overall and disease-specific survival). An advantage to our approach is the simplicity and apparent reliability for conditions such as MI (characterized by occasional repeated events and moderate survival) and for populations such as Ontario (with moderate migration). The DisMod model is another simple way to estimate disease prevalence and appears reasonably reliable. Most disease models, whether they are simple or complex, do not uniquely identify people in a registry, and so are not as useful for many evaluative purposes.

## Conclusion

Health administrative data, such as hospital admission data, are increasingly used to estimate the occurrence of disease events. They have also been used to estimate the incidence and prevalence of disease for conditions that have either frequent repeated events or health care encounters (such as people with diabetes) and/or have a poor survival (such as congestive heart failure).

We showed that for MI – which has neither frequent events nor poor survival – a simple method that uses hospitalization data for a modest number of years can reliably estimate MI prevalence. We currently have a registry of 90% of the people in Ontario with a previous AMI hospitalization. This registry can be individually combined with other health care data (such as prescribed medications, physician services, etc.) to evaluate health care for people with an MI, amongst other descriptive and evaluative studies.

## Competing interests

The author(s) declare that they have no competing interests.

## Authors' contributions

DGM obtained funding, designed the study, oversaw the analyses and guarantees the paper. JL contributed to the study design, performed the analyses and contributed to writing the paper. PT contributed to the study design, performed parts of the analyses, TS oversaw, contributed to the study design, oversaw the statistical and contributed to writing the paper.

Funding for the study was received from the Canadian Population Health Initiative.

## Pre-publication history

The pre-publication history for this paper can be accessed here:


